# Study Protocol: Determining Research Priorities of Young Albertan Families (The Family Research Agenda Initiative Setting Project—FRAISE)—Participatory Action Research

**DOI:** 10.3389/fpubh.2018.00228

**Published:** 2018-08-28

**Authors:** Katherine S. Bright, Carla Ginn, Elizabeth M. Keys, Meredith L. Brockway, Lianne Tomfohr-Madsen, Stephanie Doane, Karen Benzies

**Affiliations:** ^1^Faculty of Nursing, University of Calgary, Calgary, AB, Canada; ^2^Department of Clinical Psychology, University of Calgary, Calgary, AB, Canada; ^3^FRAISE Steering Committee, University of Calgary, AB, Canada

**Keywords:** family research agenda-setting initiative, pregnancy and childrearing, participatory action research, parent/knowledge user-identified research priorities, health research priorities of families, parental health concerns

## Abstract

**Introduction:** Pregnancy and childrearing can be an exciting and stressful time for new parents. The maternal-child health landscape has changed dramatically over the last few decades and research priorities need to address these rapid changes. There have been limited attempts to engage and collaborate with members of the public to develop research priorities for families who are expecting or parenting an infant to age 24 months. The work that has been completed has attempted to identify parental preference for information delivery and barriers to uptake of parenting programs but has not investigated parental research priorities.

**Methods:** In collaboration with provincial research units and strategic clinical networks (SCN), we will use principles of participatory action research (PAR) as our theoretical framework/method, and a modified James Lind Alliance priority setting approach to prioritize a list of research questions that parents/knowledge users believe will support the health of their families. This will result in a top 10 list of parent/knowledge user-identified research priorities. This project will consist of three phases. In the first phase, we developed a steering committee of parents/knowledge users, healthcare providers, community agencies, and researchers to design a survey about health priorities for families. In the second phase, we will distribute the survey to diverse groups of parents/knowledge users/providers and hold a series of meetings to identify and prioritize potential questions from new parents about health issues from conception to age 24 months. In the third phase, we will collaboratively disseminate and translate findings.

**Discussion:** This study will highlight parental health concerns and recommend parent-identified research priorities to inform future research projects needed to support the health of families between conception to age 24 months. Understanding the health research priorities of families in the community will help ensure future research contributes to meaningful changes in the health of young children, parents/knowledge users, and families.

**Ethics:** This study and protocol have received ethical approved from the Conjoint Health Research Ethics Board at the University of Calgary (REB17-0014).

**Dissemination:** The top 10 research priorities will be published and additional findings from the study will be distributed through pamphlets and newsletters.

## Introduction

Investing in early childhood development has an estimated return of 800% (1). This excellent return on investment can be attributed to the critical and rapid development of brain architecture that occurs within the first 3 years (1). This crucial brain development is generally thought to result from highly complex interactions between nature and nurture (1). In other words, a child's brain development is influenced by their individual genetic code as well as the social and environmental conditions to which they are exposed. Over time, these conditions can also affect and change a child's genetic programming (2). Parents have the greatest potential to optimize social and environmental conditions that foster optimal child development (3). Supporting parents, as well as health and social services, to translate this potential into environmental conditions that foster healthy child development is critical to helping children attain their full potential for healthy outcomes (4). Engaging in collaborative research processes with families and clinicians, in local community environments, is one strategy for optimizing child development.

Despite the widely acknowledged importance of optimizing child development, researchers and service providers encounter difficulties in effective translation and implementation of research on child health and development, which is evident in the lag of practice and policy (5). This lag may be exacerbated by continually evolving maternal-child and parenting landscapes, which are influenced by cultural and environmental factors such as high parental expectations, intensive parenting, and increased pressure to perform as a parent (6, 7). As the knowledge base for supporting healthy developmental outcomes grows in breadth and scope, effectively prioritizing research investment is crucial to maximize impact and minimize research waste (8). Innovative, community-based, interdisciplinary research can substantially improve public health through including real-world multicultural settings, with relevance to the population being researched (9).

Understanding early childhood developmental and health research priorities of knowledge users, particularly parents and service providers, is vital to conducting research that is more likely to contribute to meaningful changes in health outcomes (10, 11). Engaging end users (or those who will actually use/be affected by results of the research) in research priority setting initiatives is rapidly being recognized as an effective and ethical means of prioritizing the allocation of limited public research funds (12). Not only does this promote researcher accountability, but this integrative approach to knowledge translation may reduce the lag time between producing and implementing knowledge (13).

Priority partnership initiatives focused on developing research priorities for preterm birth (14), miscarriage, and stillbirth (15) exist in the United Kingdom. Canadian health research funding bodies and organizations are including patient-oriented research as key components of both Federal—Canadian Institutes of Health Research (CIHR) and Provincial—Alberta Health Services (AHS) strategic plans.

While several Canadian research groups are leading this new partnership approach (16, 17), a priority setting approach has not yet been used with families in a community-based setting. Instead, in the province of Alberta, Canada, research projects engaging broad groups of parents have focused on identifying knowledge gaps (18, 19) and use of parenting services and programs (20, 21). While these projects used collaborative approaches with health and social services representatives, they only included parents as participants. Still, these projects provide an excellent foothold to launch engagement and collaboration with parents and caregivers, creating requisite foundations for partnership priority setting initiatives.

**Figure 1:**
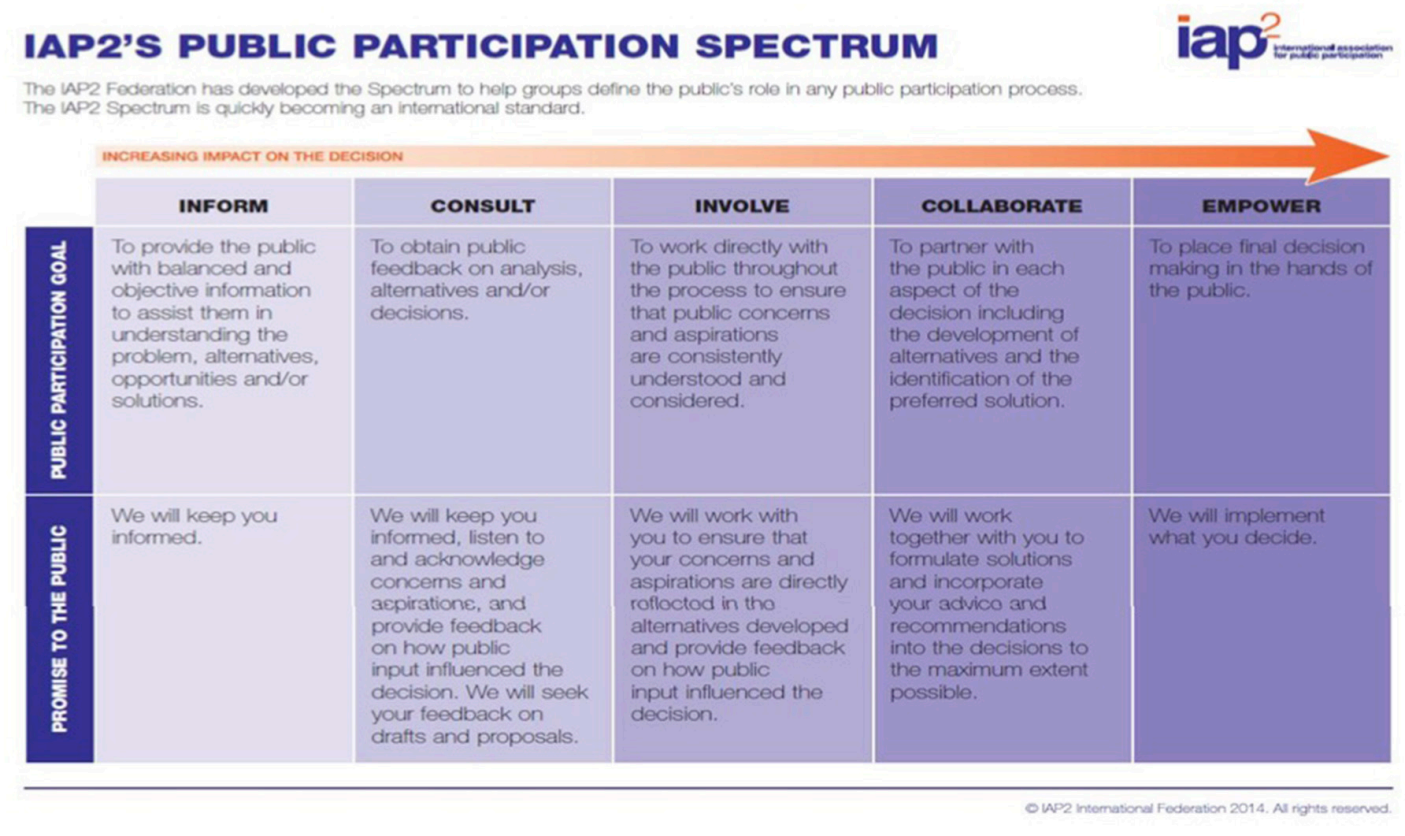
International Association of Public Participation (IAP2) public participation spectrum.

Identification of individual needs and preferences within this target population is needed to prioritize areas of concern that may occur for families who are expecting or parenting an infant to age 24 months. Parental involvement in research will enhance engagement with, and uptake of, interventions and services that support early childhood health, and may result in services that are more effective in less time. Identifying and prioritizing the needs of those directly influenced by the research (in our project, parents of young children/knowledge users) provides potential to improve research relevance. Throughout this study, participants are referred to as parents/knowledge users. We anticipate that as parents/knowledge users participate in this research project, identifying areas of research that are important to them, there will be potential for increased engagement with research and health services, contributing to improved child health outcomes. As we work with parents/knowledge users in our community to develop an understanding of their health research priorities, we will together contribute to meaningful change in priorities of future research. In the context of our research, it is important to acknowledge that individuals participating in decision-making have varying levels of influence. The International Association of Public Participation (IAP2) developed a spectrum to assist in defining the public's role in decision-making processes. (Figure 1). As individuals move to the right, they are more empowered and able to influence decisions.

This Family Research Agenda Initiative SEtting (FRAISE) project is an innovative translational and transformational research initiative that will provide a venue for public stakeholders (i.e., families who are expecting or parenting an infant to age 24 months) to voice their perspectives on research that is most relevant to their own families, and that they anticipate will positively impact their own families, communities, and subsequently, their experiences in the healthcare system.

### Aims and objectives

The overarching aim of the FRAISE project is to work with parents/knowledge users to create a prioritized group of research questions that families, in collaboration with researchers and service providers, believe will support their health and wellbeing. This research will help identify strategies that contribute to sustained engagement of parents/knowledge users with healthcare providers, as well as researchers, ultimately supporting healthy outcomes for children and families. The long-term goals for the FRAISE project are to: (1) increase access to stakeholder and parent/knowledge-users population in terms of child and family health; (2) decrease lag in research to practice/policy development; (3) improve child and family health outcomes; (4) decrease research waste; and (5) infuse Patient Oriented Research (POR) into family health research topics and outcomes.

## Methods and study design

### Study design

Researchers with the FRAISE project will apply a consensus-building and strengths-based approach, modeled after participatory action research (PAR) and a priority setting model (10, 11). Through the writings of Dewey (1859–1952), Collier (1884–1968), Lewin (1890–1940), and Freire (1921–1997), PAR emerged as a theoretical perspective in which to frame research, as well as a method to engage in research. Priority setting partnerships bring together patients, caregivers, and clinicians, requiring sensitivity to varying capacities, ongoing effective communication, transparency in decision-making, and inclusivity of all views (22). The FRAISE project uses a modified priority setting method (22) with a three-phase design, including parents/knowledge users and key service providers who receive and provide care within the local and provincial health authority.

PAR entails engaging with a community who have self-identified a topic of common interest or problem; priority setting involves engaging with patients, caregivers, and service providers with commonality of a condition. On an international level, priority setting partnership research has addressed a wide range of health conditions including cancer (23), mental health (24), women's health conditions (25), and musculoskeletal diseases (26). Examples of current Canadian priority setting partnerships include head and neck cancer (27) and hypertension management (28). Another Canadian study with dialysis patients and caregivers (16) outlined a priority setting process that included: (a) formation of a steering committee, including patients and clinicians; (b) entering priority setting partnerships as identified by the steering committee; (c) gathering research uncertainties or questions; (d) categorizing or grouping questions; and (e) determining the top 10 research priorities (with patients, caregivers, and clinicians in full-day workshops). After theming research topics/priorities, systematic literature assessments are used by the research team and interested members of the steering committee to define well-, under-, and un-researched topics.

**Figure 2:**
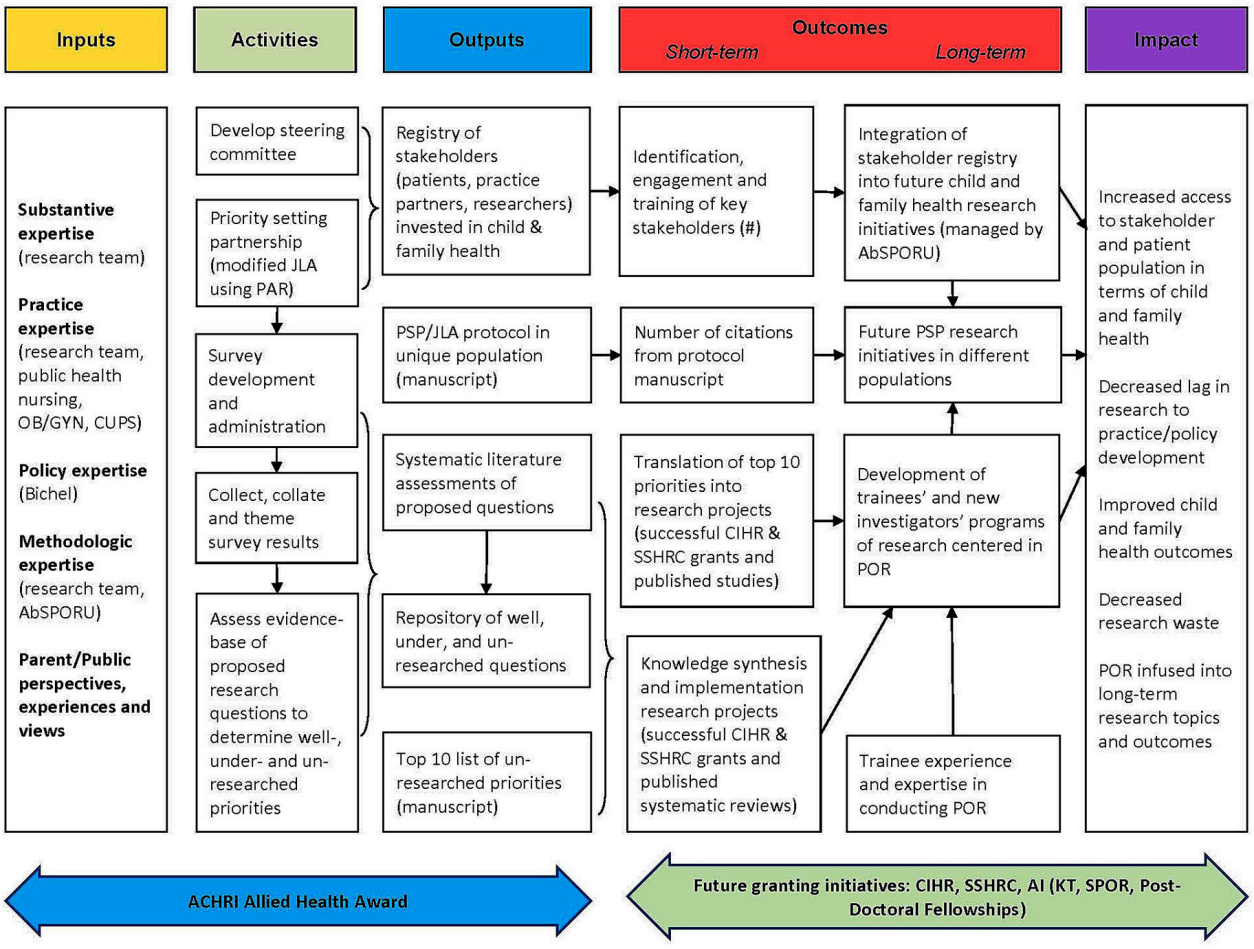
Family Research Agenda Initiative Setting (FRAISE) program logic model.

In priority setting research, determining the priorities of vulnerable groups remains a challenge for the priority setting process (16); as PAR is grounded in emancipatory research with vulnerable groups, utilization of its principles are most fitting. Challenges in priority setting projects include developing equitable patient and professional interactions; recommendations for addressing reciprocal relationship building and processes of co-learning, including researcher training in participatory methods (29). While remaining flexible and open to emergence of new ideas using PAR, the FRAISE research project adheres to an overarching framework. This framework has three phases. Phase I involved the formation of a steering committee. Phase II will involve the setting of research prioritization partnerships, and Phase III, analysis and dissemination. These three phases are part of the broader FRAISE project outlined in the FRAISE program logic model (Figure 2).

### Phase I: formation of a steering committee and survey development

We are currently in Phase I of our three-phase study. Considering how important it is to understand the wants and needs of our target population, we considered the formation of a steering committee as the most appropriate and informative approach for gathering these perspectives. This committee was formed in October 2017 and includes parents/knowledge users (new parents from conception to age 24 months), committee members, community agency representatives, and researchers. Our aim is for 50% of the champions to be families, with 30% of them experiencing vulnerability. We are aiming for survey distribution to mirror the Calgary population, including ~3% of Calgarians who are First Nations, Metis, and Inuit and 30% who are immigrants and/or visible minorities (30).

#### Steering committee membership

We are using a two-pronged approach for recruitment of steering committee members. The core research group coordinated a broad social media campaign alongside a more targeted purposeful outreach. The broad social media campaign was designed to capture a wide-breadth of members of the public whose profiles indicated they had interests in pregnancy, infancy, or toddlerhood. Other potential steering committee members were approached using a targeted outreach strategy, according to representativeness and capacity to provide rich and varied information from health/social-service boards, networks, and agencies. When describing steering committee involvement, we clearly explained to potential steering committee members that their knowledge and quality of parenting/clinical practice would not be evaluated. We explained that rather than researching their own parenting or clinical abilities, this research project was about gaining understanding of areas of need, or gaps in knowledge affecting young families and healthcare providers invested in their care. It was anticipated that the steering committee would consist of a total of 25–30 members from variety of backgrounds to try and increase diversity, essential to promoting discussion and interactions among members. Steering committee members were only required to provide a signed confidentiality agreement and not consent forms. Our steering committee currently has 21 participants, including 7 mothers, 7 clinician and community partner or parent/knowledge user representatives, and 7 child health researchers.

#### Planning and structuring steering committee activities

The first role of the steering committee will be to design a survey about health priorities for families. The steering committee met together with FRAISE researchers as a group where they engaged in patient engagement training sessions though the Alberta Strategy of Patient-Oriented Research Support Unit (AbSPORU) patient engagement platform. Together with the AbSPORU, we engaged in reciprocal relationship-building exercises as a steering committee. The AbSPORU provided guidance from the project inception through the steering committee formation and has continued to provide ongoing support in this research process. POR involves capacity-building in both researchers and participants to develop a common goal of developing, discovering, and disseminating knowledge. Together members determined parent engagement strategies of how best to “ask the questions”. We are currently in the process of developing a survey to explore research uncertainties and questions of parents/knowledge users, families, and clinicians.

#### Steering committee meeting moderating

AbSPORU and the research team moderated the initial steering committee meetings (held over 1.5 days). Subsequent committee meetings will be designed to be flexible and open to the topics and issues raised by all members, with a combination of in-person and online approaches. The research team met following the initial steering committee meeting to collate the experiences related by participants, and the information collected. This information was analyzed and themed. During the steering committee meeting we collaboratively developed the next steps for the committee including establishing: (1) roles associated with designing and implementing the survey as well as committee members intersted in the role of “championing FRAISE and the survey” to help increase uptake; (2) roles that are covered in different elements of the project, i.e., survey and data collection, analysing and theming, prioritizing, and dissemination; (3) how the steering committee will stay connected by exploring preferences for virtual or in-person meetings based on what stage/phase the project is in; and (4) frequency of steering committee meetings while finalizing the survey, once the survey has been rolled out, and once there are results from the survey.

#### Data organization and validation

Together with the AbSPORU team, the steering committee developed 12 draft themes using a consensus-building approach about their concerns that will inform a subsequent survey developed for distribution in Alberta, Canada, to uncover parent-identified research gaps.

The draft themes identified include:

- Infant and/or toddler feeding
- Stress, emotional and/or mental health
- Sleep (infant, child, and parents)
- Vaccinations (for babies, children, and parents)
- Social, family, and intimate relationships
- Labour and birth experience
- Parenting confidence
- Keeping your baby safe enough
- Growth and development
- Environmental risks for myself and my child
- Accessing health information (where do you get your information and what has been helpful/not helpful)
- Child care.

In addition, the research team and AbSPOR team gathered suggestions from the steering committee for moving forward in building and disseminating the survey:

- Identified individuals/groups who should complete the survey - including a list of target groups (i.e., pregnancy, post-partum, toddlers)
- Identified additional groups and agencies our steering committee needs to connect with including a list of agencies (i.e., Families Matter, Parent Link Centres, Primary Care Networks, SCN, La Leche League)
- Explored options for accessing target populations and groups including utilization of virtual/social media networks
- Explored methods for delivering the survey including opinions for online, tablets, paper, and pen
- Explored ideas for advertising/recruitment (i.e., specific social media groups to access)
- Identified options for encouraging recruitment of both mothers and fathers to participate as well as ways for achieving diverse representation from parent/knowledge user participants.

All information generated from the steering committee will be documented and digitally recorded. This information will be digitally stored on a protected drive. The information obtained will be analyzed, themed, and patterns will be identified, keeping in mind the text and the context in which the ideas have been produced. Steering committee members who wish to be part of the process of data analysis will be invited to do so. The process and provisional analysis will be combined, consensus will be sought among the investigators, and the results will be formatted into the development of a pilot survey.

### Phase 2: survey and priority setting partnership

#### Design of the survey

We will use a single questionnaire divided into two main blocks. The first block will consist of the 12 previously determined themes by the steering committee that evaluate parent/knowledge users', healthcare providers', community agencies' to views of health priorities for families. An additional question will assess if survey participants have any other questions that did not fit into the 12 previously determined themes. We will contrast the original structure and items on the survey with the information obtained from the steering committee meeting to refine and complete its content and make it more representative of our local environment. The second block of the survey will gather demographic details of participants.

#### Pilot survey

Upon completion of the draft, we will distribute the survey to the steering committee again for face and content validity. Steering committee members will be invited to provide feedback on the content of the items as well as flow, ease of use, and appropriateness of stems. The steering committee will achieve consensus on the final survey prior to distribution to a broader audience, new parents from conception to age 24 months throughout Alberta, Canada.

The survey will also be piloted prior to the main rollout of the survey. Parents of both sexes who meet inclusion criteria for this study will be selected to evaluate the comprehension, feasibility, and length of the questionnaire.

#### Recruitment and sample

The survey will be distributed using several modalities, including online, open forums at community sites, and hardcopy distribution at outreach sites to ensure a broad and diverse sample. Investigator contact details will be provided for participants to raise questions or inquire about the questionnaire or the project.

Based on the number of respondents in previous priority setting studies, we estimate a sample size of 500 respondents is needed to address the main objective of the survey (to increase access to stakeholder and parent/knowledge user populations in terms of child and family health). This sample size estimate is in line with recent priority setting research: 550 participants (78% patients and their caregivers) identified the top 10 questions for researching irritable bowel disease in the UK (31), while 413 participants (51% patients and their caregivers) identified the top 10 questions for endometrial cancer research (32). Our estimated sample size is also consistent with sample sizes from Canadian priority setting partnership research: 583 participants identified research priorities for Type 1 diabetes (33) and 438 participants identified research priorities for patients with chronic kidney disease not on dialysis (34).

#### Committee engagement opportunity

Parents/knowledge users who completed the survey will be invited to select additional engagement options, such as joining a participant registry, participating in the prioritization workshops, and/or joining the steering committee.

### Phase 3: collaborative analysis and dissemination

There will be a series of forum-style sessions to theme and prioritize research topics identified in the survey. After an initial session to theme research topics, the steering committee will conduct systematic literature assessments to define well-, under-, and un-researched topics. During these sessions, the committee will prioritize the top 10 under and un-researched topics emerging through consensus-building processes. All members of the steering committee will be offered opportunities to participate in the collaborative analysis and dissemination stages of the research project.

#### Identifying research areas

Questions identified through the survey entries will be grouped into themes. Steering committee members will be asked to analyse each theme to determine inclusion or exclusion to the priority setting. Previous priority setting studies have required 2 steering committee members analyse one theme (one patient and one clinician) to minimize researcher bias (32). Discrepancies between the reviewers will be settled through independent review.

Each theme will be searched against the literature and those that have already been investigated will be held in a repository for future KT strategies. Well researched themes will be defined as those that have “recent level 1 evidence (systematic review within last 3 years)” (32).

#### Prioritizing research areas

Questions that have been asked by at least 25% of survey respondents will automatically be moved forward to the face-to-face prioritization round. Questions that were asked by 3 independent respondents in the “other” question will also be themed and moved forward to the prioritization round.

Over a period of ~8 months, the steering committee will meet regularly for pairing down the 30 important uncertainties and agreeing on a top 10 list of research uncertainties and questions of parents/knowledge users, families, and clinicians. In the face-to-face prioritization round, the steering committee will be divided into three groups with equal representation from parents/knowledge users, clinicians, community agencies, and researchers in each group. A modified nominal group technique facilitated by the AbSPOR will be used to rank uncertainties. Rankings will be aggregated and the three groups within the steering committee will be redistributed to review the aggregated list of ranked priorities. Considering this list of weighted ranks, the steering committee will reconvene and agree on a final set of 30 important uncertainties to be considered at the final prioritizing meeting.

#### Top 10 research areas

A final consensus meeting will be held with the steering committee members to determine the top 10 research priorities. If we cannot reach a consensus of 10, we will allow slightly more according to the consensus of the group. This final meeting will be facilitated by the AbSPOR to ensure equal participation and contribution by all steering committee members. Consensus building will occur through similar priority setting techniques and discussions as previously mentioned. It is anticipated that the final top 10 research priorities will demonstrate the breadth of research required to better support the health and wellbeing of families.

#### Knowledge translation and dissemination

This project will create a foundational integrated knowledge translation (iKT) framework for a publicly informed research agenda of community-based maternal and child health research topics. Our knowledge translation plan is informed by the Knowledge-to-Action cycle (35, 36) and the spectrum of public engagement developed by the International Association for Public Participation (37). We have three broad knowledge translation goals: (1) sustained engagement of key stakeholders throughout the project; (2) dissemination of the prioritized health priorities; and (3) translation of the research priorities into actual research projects.

1. **Sustained engagement of key stakeholders**. We have identified three target audiences to engage the steering committee, public/parents, and researchers. We have already begun to engage with stakeholders and potential knowledge users to build awareness, collaborations, and partnerships. This includes the creation of a stakeholder registry, a dynamic document that includes each potential knowledge user or stakeholder and their level of readiness to engage.
  - It is essential we create sustained engagement with the core group of the steering committee. To engage members of the steering committee, which will include researchers, parent champions, service providers, and community agency representatives, we will have face-to-face meetings that accommodate the presence of children, interspersed with email, online, and tele-communication. We will vary the location of our steering committee meetings within community venues to ensure they are accessible to different members. We will assign a specific team member to provide regular updates and engagement activities for the steering committee.
  - To engage parents and other members of the public, we will use Facebook, an acknowledged medium to communicate with and engage parents (38–40). We will also use community agency representatives on the steering committee to act as knowledge brokers to engage parents within their respective catchments (i.e., CUPS Health Education Housing and the Ethno-Cultural Council of Calgary).
  - To engage researchers beyond our immediate team, we will network with specific research groups, such our upcoming meeting with the Maternal Fetal Standing Committee, and use strategic networking at patient-oriented or child health conferences (ACHRI symposium, AbSPORU Summer Institute, KT Canada Summer Institute). In addition, each research team member will use their established connections with existing internal and external research groups (Canadian Child Health Clinician Scientist Program, ACHRI Healthy Outcome Rounds, Research and Innovation for Population, Public and Indigenous Health [AHS]) to build awareness and engagement. Dr. Allison Bichel, AHS provincial lead for the Maternal, Newborn, Child and Youth and the Addictions and Mental Health SCN, as well as the respective scientific directors (McNeil and MacMaster), are collaborating with the research team to align this research with the SCN and AHS research priorities (letter of support attached). Our research strategy is aligned with the recently prioritized *demand-pull system* to determine healthcare innovation within Alberta Health (Justin Reimer, Assistant Deputy Minister, Alberta Health, personal communication, March 6, 2017).
2. **Dissemination of research priorities**. The PAR approach uses an iKT strategy of involving participants in theming research priorities. Our group will further extend this iKT strategy by generating opportunities for parents to co-create knowledge products to be used for dissemination. Depending on parental readiness to engage and their desired level of engagement, there will be opportunities to participate in manuscript preparation, conference and rounds presentations, as well as development of non-academic products that could be distributed to lay audiences (e.g., magazine or online articles, blogs, infographics). Our **sustained engagement** with our target audiences will assist in this process.
3. **Translation of research priorities into research projects**. Our final knowledge translation goal focuses on ensuring the prioritized health research topics are translated into actual research projects, including projects funded by CIHR Knowledge Translation Grants. At a minimum, this project will form the foundations of sustainable child and family-centerd research programs by informing the future research of our outstanding and emerging academic team. To support researchers in addressing the prioritized research topics by successfully obtaining funding for related research projects, we will conduct rapid reviews of the literature on the specific research priorities. We will publish these reviews as a tool for researchers and organizations such as CUPS Health Education Housing to facilitate grant and research funding applications. To assess impact of this research project, we will collect indicators on the number of grant applications that address the prioritized research topics, along with the success rates of these applications.

## Ethics and dissemination

### Ethical considerations

Ethical considerations for conducting this study were based on a comprehensive thematic literature review exploring ethical community-engaged research (41). In our study, all partners will be equally included in the project at all stages, with participant and community values and expectations understood. We aim to prioritize transparency with open, honest, and continual communication. Additionally we developed our steering committeee for developing research questions and interpreting data. The Conjoint Health Research Ethics Board at the University of Calgary have reviewed and approved this study. This study will promote professional/ethical development through reflexive research ethics including reliance on professional morality, continual reflection, and cultural humility. This study will maintain a rigorous POR research design.

### Informed consent and institutional review boards

This protocol and study, including the informed consent forms, have received ethical approved from the Conjoint Health Research Ethics Board at the University of Calgary (REB17-0014) and will be reviewed annually.

In all cases, following a comprehensive explanation of the purpose of the study in the online consent form, participants will be provided with information regarding the project's objective and the participant's role in the study. Individual informed consent will be obtained from each participant by ensuring they understand the study purpose, what partipation in the study entails, and potential risks and benefits of participating in the study. Consent forms will be written at the 8th grade reading level. Consent to participate in the survey is thereby established upon acceptance of participation. Participants are given the option to withdraw from the study at any time. For participants who may experience distress following their completion of the survey, the survey will a link to a list of Alberta wide community resources. Additionally, the FRAISE Project webpage will also include the Alberta wide community resources.

### Confidentiality

The instruments used is a self-administed anonymous survey in which the participants are not requested to give their full names. Only their first name, first three digits of their postal code, age of children, and ethinicity will be registered in the database. For participants who wish to be receive the $5 gift card for completing the online survey, they will be asked to provide their email address.

The survey data will be housed on a timed out, password protected computer, in a secure room at the University of Calgary campus. Access to this data will be restricted to the researchers directly involved in the study. The database linking codes for participant numbers and their confidential information (name and email address) will be kept seperately from the actual survey contents. Back-up data files will be stored in locked filing cabinets in a separate room on password protected hard drives.

### Dissemination

The research team anticipates publishing one primary paper that reports the top 10 research priorities of parents/knowledge users, families, and clinicians. Additionally, the top 10 research priorities will be shared across traditional academic discipline boundaries in a manuscript with future recommendations to encourage creative and independent approaches for addressing the same issue. Findings from the study will be distributed through pamphlets and newsletters and presented at meetings to local health care practitioners and agencies who supported study recruitment through a final report and to the participants themselves. Research uncertainties already investigated but requiring improved KT strategies for disseminating this information will target individuals during the childrearing years, community providers within public health departments, community agencies, and policy makers including early childcare providers, social workers, psychologists, registered nurses, physicians, program managers, coordinators, or directors. KT will focus on two areas, change in knowledge and change in practice, and will involve face-to-face communication interventions, health-care provider training, community-based actions, and communication using mass media.

Following study completion de-identified data will be submitted to Secondary Analysis to Generate Evidence (SAGE), a research and data facility operating under the authority of Policywise for Children & Families (https://policywise.com/initiatives/sage/). SAGE stores high-quality research, community service, and administrative health and social well-being data. Repositing our data with SAGE will ensure that more knowledge will be generated from the information from our study. SAGE works to promote knowledge mobilization and document the impact of secondary data uses.

## Discussion

### Anticipated results

This project will have two key areas of impact: (1) to involve and include parents/knowledge users and clinicians in health research; and (2) to inform future research initiatives. By providing a venue for parents/knowledge users and families to voice their opinions on what research is needed to support the health and wellbeing of new parents from conception to age 24 months, we will develop improved strategies for parents/knowledge users to address their perceived barriers and preferences for information and care. It is anticipated that this research will create more acceptable services for families including innovative access and evidence-based treatment options that enable more equitable access to services across the province. Further, involving practitioners in the priority setting process will enhance buy-in and implementation of new evidence-based practice recommendations. These recommendations, combined with a decreased lag time from research to practice, will result in improved child and family outcomes.

The second area of impact will be evident in future research initiatives structuring programs of research around the top 10 research priorities to ensure that their emerging research activities encompass identified priorities of parents/knowledge users. Finally, repositing well- and under-researched priorities will establish a database for parent/knowledge user informed future knowledge synthesis and KT implementation research projects.

### Limitations

This innovative approach to parent/knowledge user engagement presented several challenges at the steering committee phase, before survey development and distribution to participants. One challenge involved our agreement with a professional nanny service. As the date for the steering committee neared, they canceled their booking. We were faced with the potential of 30–40 children with no child care. Our solution to mitigate this challenge included purchasing extra toys and floor mats for the area of the meeting room divided off for child care. In addition, one of the research team members enlisted her licensed day home provider who quickly mobilized her team to on-site child care at the steering committee meeting. We had informed the parents there would be on-site child care, which was central to their ability to attend. A second challenge was that during the steering committee meetings many of the mothers expressed feeling very tired. Our solution to partly mitigate this challenge was to offer frequent breaks and meals/refreshments throughout the 1.5 days. We also considered carefully the participant burden presented by the in person steering committee meeting, and future meetings, holding our next meetings via social media and online. A third challenge we noted was, although we had purchased taxi chits (vouchers) for mothers without transportation, none of them were used. Our solution to partly mitigate this challenge was speaking with our community partners who provide services for vulnerable members of our community, and realizing the necessity of spending time at these community organizations. We now anticipate that emailing a survey will not be an effective means of data collection for those accessing these agencies. We plan to have our trained research assistants visit the community organizations, give parents who volunteer and consent access to the survey via ipads and sit with them, answering questions as they engage in completing the survey we are developing.

## Conclusion

We anticipate that project outcomes will impact both parents/knowledge users and the healthcare system by providing a venue to voice parental opinions on what research is needed to support the health of families from conception to age 24 months. This project aligns with the priorities outlined by national health funding research bodies (Canadian Institutes for Health Research, National Institutes of Health, and the National Health Service) because we will identify individual needs and preferences of the target population by prioritizing health problems and making recommendations for research strategies that address parent/knowledge users' needs. This project will result in sustainable child and family-centered research programs by informing the future research projects of child health researchers on a local, national, and international scale. Understanding the health research priorities of families in the community should ensure future research contributes to meaningful changes in the health of young children, parents, and families. Through identification of the top 10 research priorities for young families, this research will contribute to research methods and developing interventions with potential to promote the health and wellbeing of children and families.

## Authors contributions

All authors, KSB, CG, EK, MB, LT-M, SD, and KB, contributed significantly to the concept and design of the manuscript. All authors critically reviewed and provided feedback on the final revised version submitted for publication. KSB and CG, the first two listed authors, have contributed equally as first author to the protocol.

## Permission to copy, reproduce, or publish

Permission was obtained to copy, reproduce, and publish the International Association for Public Participation (IAP2) Spectrum of Public Health from the IAP2 on behalf of the IAP2 International Federation.

### Conflict of interest statement

The authors declare that the research was conducted in the absence of any commercial or financial relationships that could be construed as a potential conflict of interest.
